# Antepartum Thoracocentesis: A Case Report on Congenital Chylothorax

**DOI:** 10.7759/cureus.90275

**Published:** 2025-08-17

**Authors:** Maria Almeida, Marta Campos, Celia Soares, Cristina Godinho, Maria Brito

**Affiliations:** 1 Obstetrics and Gynecology, Unidade Local de Saúde de Trás-os-Montes e Alto, Vila Real, PRT; 2 Obstetrics and Gynecology, Unidade Local de Saúde de Gaia e Espinho, Vila Nova de Gaia, PRT

**Keywords:** congenital chylothorax, fetal pleural effusion, obstetrics ultrasound, prenatal diagnosis, thoracocentesis

## Abstract

Congenital chylothorax remains a poorly understood condition, and the optimal approach to its management continues to evolve. Rapid progression of pleural effusion with mediastinal shift and/or development of hydrops fetalis are key indications for urgent fetal intervention to enable thoracic decompression and prevent fetal compromise. We report a case of isolated mild unilateral pleural effusion identified during routine midtrimester ultrasound, successfully managed through close ultrasonographic surveillance and perinatal thoracocentesis performed immediately before delivery to facilitate lung expansion and minimize cardiopulmonary compromise. Prenatal intervention in this context was associated with favorable neonatal outcomes, including improved Apgar scores, reduced ventilator dependence, and fewer complications.

## Introduction

Fetal pleural effusion is characterized by a nonspecific accumulation of fluid within the pleural space, which may present unilaterally or bilaterally [1]. It is estimated to occur in approximately 1 in 10,000 to 15,000 pregnancies [2] and exhibits a male predominance with a reported male-to-female ratio of 2:1 [3].

Pleural effusions are classified as primary or secondary according to their underlying etiology. Primary effusions, or hydrothorax, referred to as congenital chylothorax postnatally, are typically caused by lymphatic fluid accumulation in the pleural space due to thoracic duct leakage, excessive lymph production, or impaired lymphatic drainage [2,4]. Secondary pleural effusions are commonly associated with fetal structural defects, such as congenital cystic adenomatoid malformation, bronchopulmonary sequestration, congenital diaphragmatic hernia, mediastinal tumors, or congenital goiter, with genetic abnormalities including trisomy 21, X monosomy, and Noonan syndrome. They may also arise in the context of congenital infections such as toxoplasmosis, syphilis, varicella, parvovirus B19, HIV, rubella, cytomegalovirus, and herpes simplex (TORCH), or in association with immune and nonimmune hydrops [2,4,5]. Fetal anemia should be ruled out during the diagnostic workup [2]. Pleural effusions are most frequently detected during the second or early third trimester [1]. Thoracocentesis, performed at the time of amniocentesis, may be useful for diagnostic clarification [6]. Comprehensive prenatal evaluation is crucial to determine the underlying etiology, guide prognostic counseling, and develop an appropriate management strategy [7].

Given the variable clinical course of congenital chylothorax, which may range from spontaneous resolution to progressive deterioration leading to cardiac failure and hydrops, clinical decision-making regarding intervention, premature delivery, or expectant management can be challenging [2]. In severe cases, pulmonary hypoplasia may result in neonatal death [4,8,9]. Prenatal management strategies for fetal pleural decompression vary across perinatal centers and may include thoracocentesis, pleuroamniotic shunting (PAS) placement, or pleurodesis using sclerosing agents [10]. Prenatal diagnosis enables timely postnatal intervention, thereby reducing the risk of neonatal asphyxia. Peripartum thoracocentesis, performed either immediately prior to delivery or just before umbilical cord clamping, is commonly performed to promote lung expansion and support neonatal resuscitation, which is particularly critical during the initial minutes of life [5,11,12].

We report a case of isolated unilateral fetal pleural effusion diagnosed in the second trimester, which was successfully managed through conservative monitoring with serial ultrasound evaluations and urgent thoracocentesis performed immediately prior to delivery.

## Case presentation

**Figure 1:**
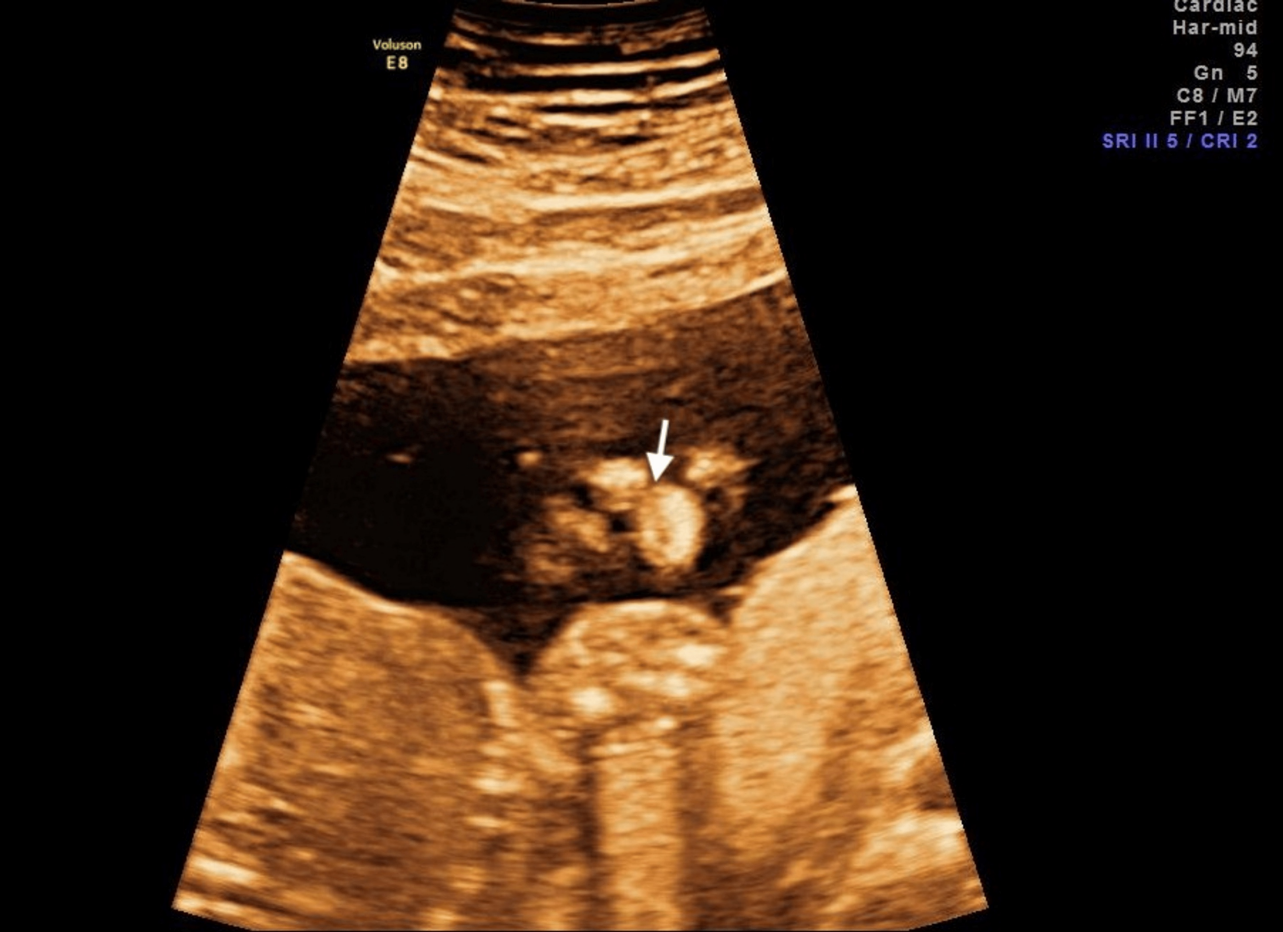
Unilateral pleural effusion at midtrimester ultrasound Right unilateral mild pleural effusion and cleft lip (white arrow) at midtrimester routine ultrasound (coronal view).

A 29-year-old Caucasian woman, gravida 2 para 1, underwent a routine midtrimester ultrasound at 20 0/7 weeks’ gestation, which revealed a mild right-sided unilateral pleural effusion and a cleft lip (Figure 1).

**Figure 2:**
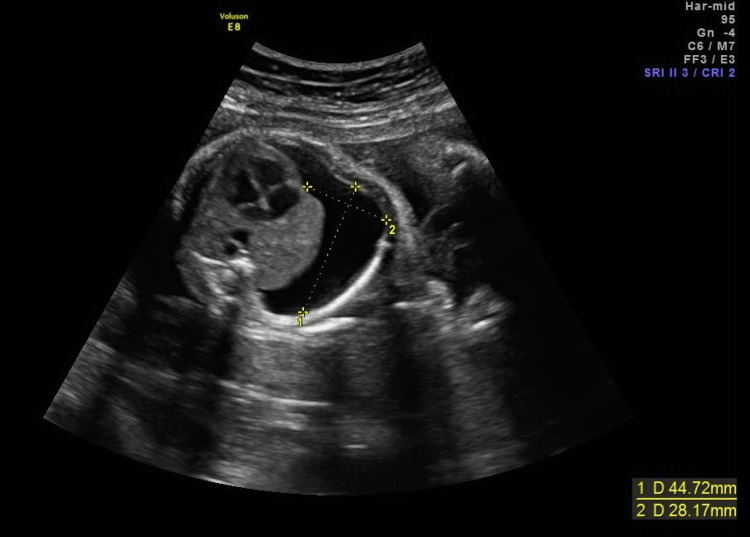
Pleural effusion at 28 weeks Axial section of right pleural effusion (44 × 28 mm) at 28 weeks.

The remaining fetal anatomical survey was normal. Her pregnancy surveillance was uneventful up to this date, with an unremarkable ultrasound at 13 0/7 weeks. Maternal serum laboratory results for viral infections (TORCH group and VDRL) were negative. Prenatal labs revealed maternal Rh-positive status with negative antibody screening. The fetal echocardiography at 20 0/7 weeks was normal. Amniocentesis was performed at 20 6/7 weeks. The fetal karyotype was normal (46, XY), and the microarray CGH was also normal. A next-generation sequencing panel for Noonan syndrome was also performed and was normal. The infectious studies, including toxoplasmosis, HSV 1 and 2, cytomegalovirus, and parvovirus in the amniotic fluid, were negative. Serial ultrasounds performed every two weeks showed a stable right-sided pleural effusion up to 28 weeks’ gestation. At that point, a significant increase in effusion size was observed (measuring 45 × 28 mm), associated with leftward mediastinal displacement, although no evidence of fetal hydrops was detected (Figure 2).

**Figure 3:**
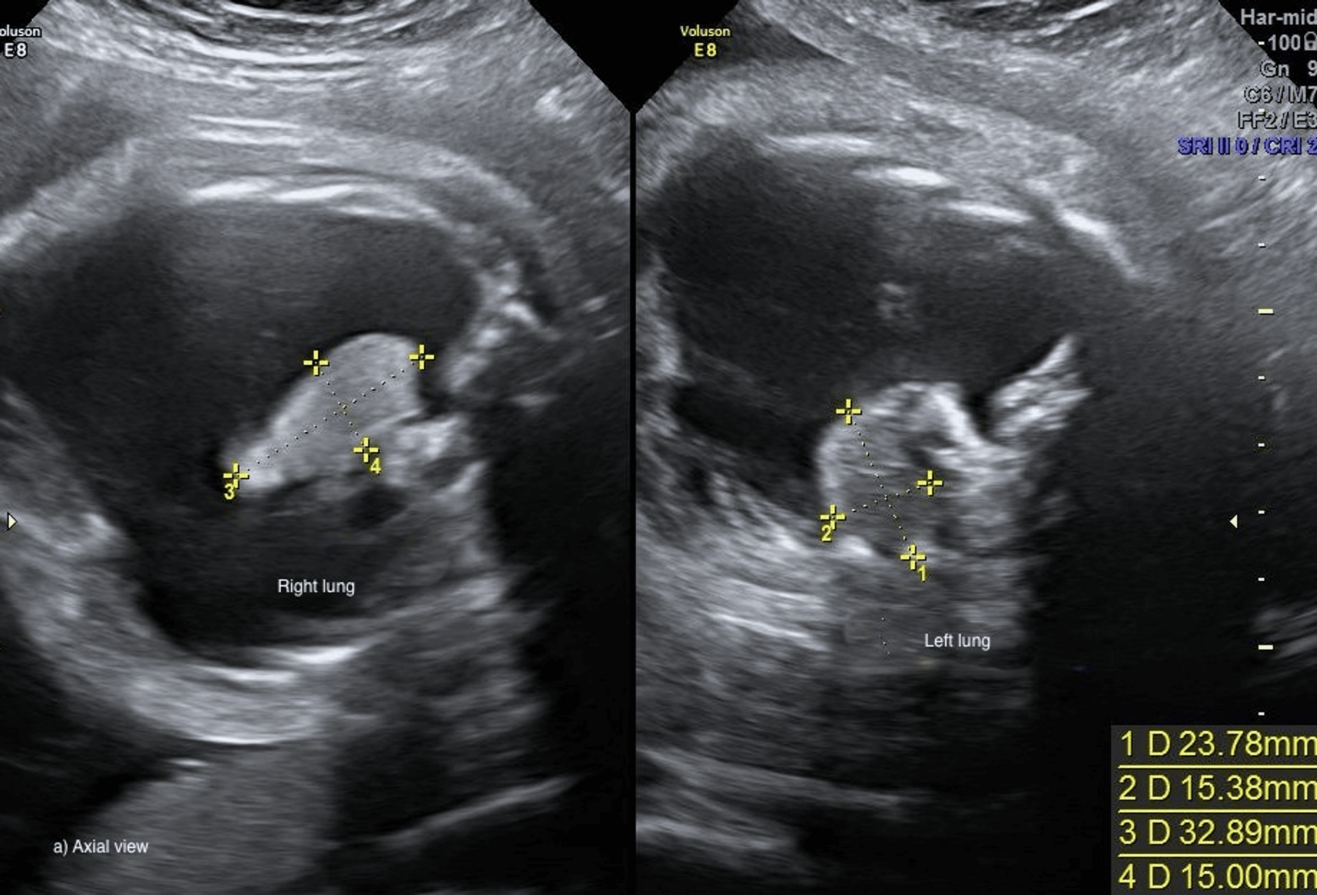
Bilateral pleural effusion, mediastinal shift, and severe hydramnios at 33 weeks Axial view of bilateral hydrothorax, with left mediastinal shift and severe hydramnios at 33 weeks (pleural effusion 55 × 19 mm).

Thoracocentesis was performed at 28 3/7 weeks after administration of a course of antenatal steroids for fetal lung maturation. A total of 45 ml of yellow-colored fluid was drained from the fetal right hemithorax. Cytological analysis revealed a white cell count of 8054/l, with 99.4% of lymphocytes, consistent with a diagnosis of congenital chylothorax. A control ultrasound performed three days later demonstrated fluid re-accumulation (right pleural effusion 59 × 18 mm, with left mediastinal shift). Fetal MRI confirmed a bulky right pleural effusion 20 mm thick along the right hemithorax associated with lung collapse and left mediastinal shift. The left lung was well expanded with no signs of effusion. The right pleural effusion remained stable throughout the surveillance, but at 33 2/7 weeks, hydramnios (amniotic fluid index of 23 cm) was observed, accompanied by rapid progression to bilateral pleural effusion and mediastinal shift (Figure 3).

**Figure 4:**
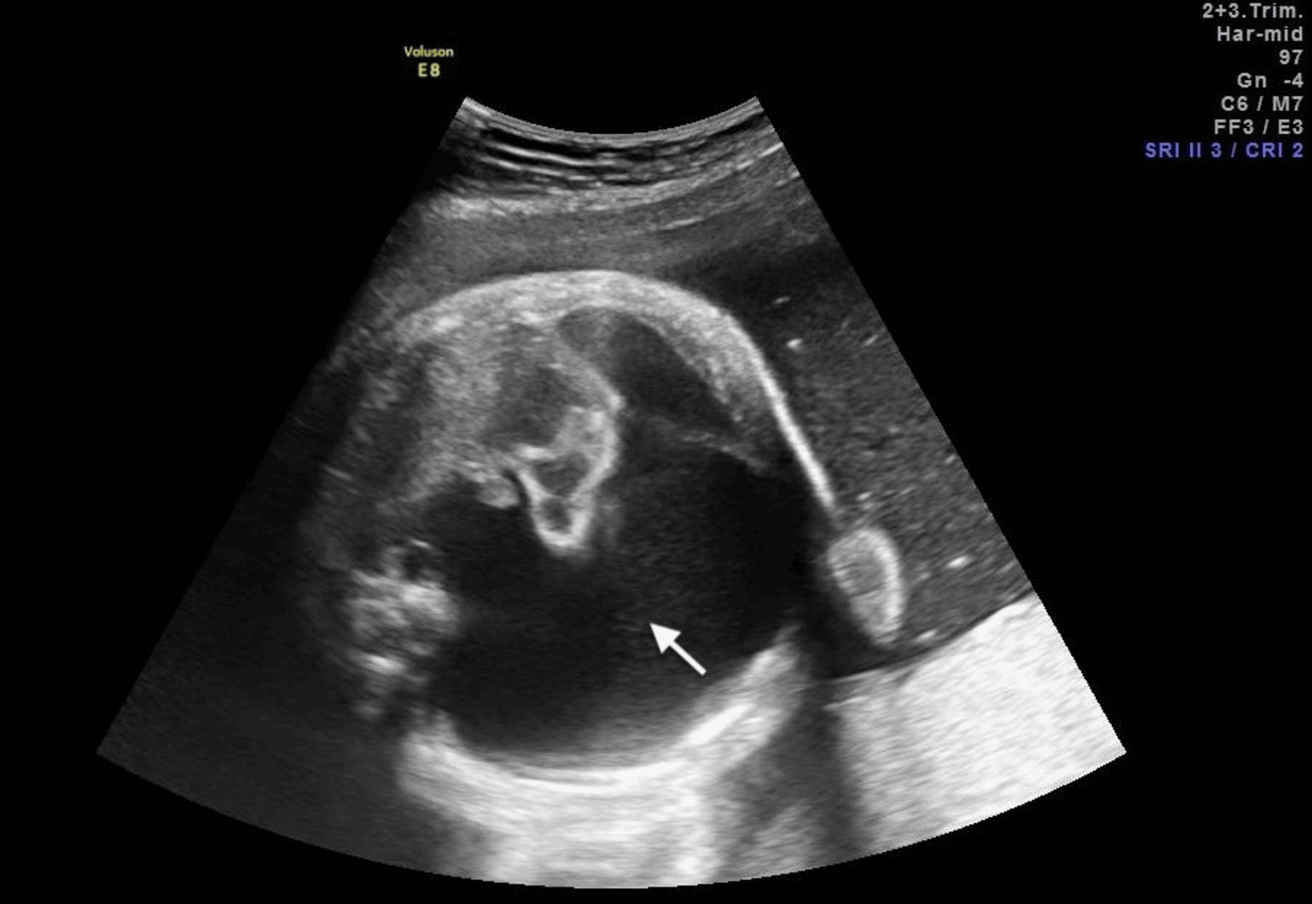
Pleural effusion at 36 weeks Large hydrothorax (white arrow) at 36 weeks.

Following comprehensive counseling on potential risks and management strategies, PAS was offered as an interventional option; however, the patient declined, and close monitoring with twice-weekly ultrasound was maintained throughout the remainder of the pregnancy. At 36 2/7 weeks, the patient had preterm premature rupture of membranes (PPROM). Fetal thoracocentesis was performed immediately prior to cesarean delivery under ultrasound guidance, with drainage of 150 mL of orange-yellow-colored fluid (Figure 4).

**Figure 5:**
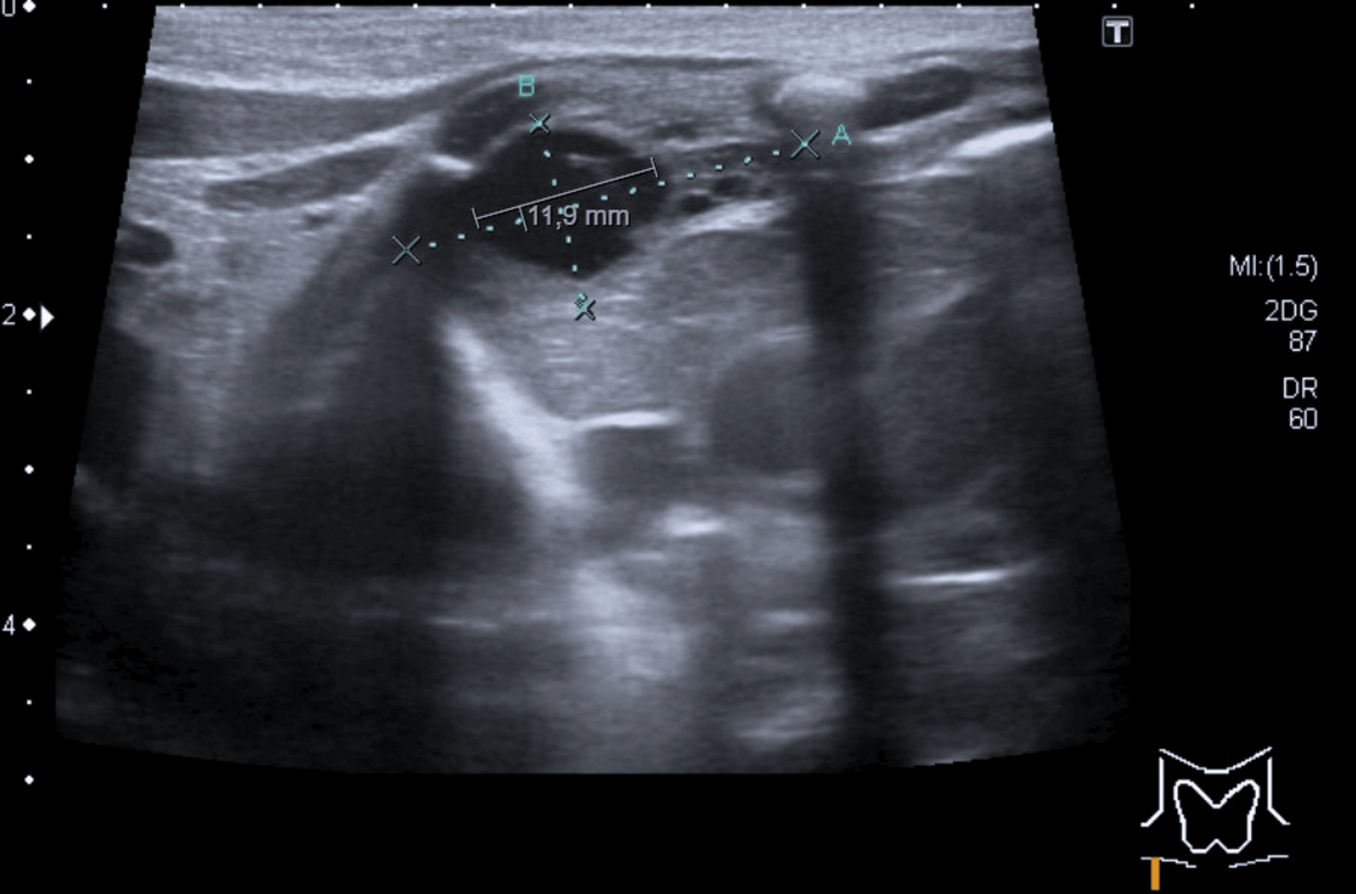
Lymphangioma in the anterior mediastinum Ultrasound image of the anterior mediastinum showing a right-lateralized, multiloculated cystic lesion (measuring 31 × 27 × 12 mm), located anterior to the thymus. The lesion is avascular on Doppler evaluation and is suggestive of a lymphangioma (axial view).

A male newborn was delivered, weighing 2696 g, with an Apgar score of 8 at the first, fifth, and 10th minute with no need for resuscitative efforts. Nonetheless, due to signs of neonatal respiratory distress syndrome, he was admitted to the neonatal ICU. On admission, invasive mechanical ventilation was initiated, and a right-sided thoracic drain was placed, yielding 50 mL of serous-hematic fluid. A control chest radiograph confirmed resolution of the pleural effusion. The characteristic lymphocytosis was found in the sterile chylous fluid. By the seventh postnatal day, the neonate was breathing spontaneously without supplemental oxygen. Due to persistent chyle drainage exceeding 10 mL/kg/day, octreotide was initiated at a continuous infusion rate of 0.5 mcg/kg/h and maintained until the 20th day of life. On day 12, the thoracic drain was removed as the chylothorax had become residual, as confirmed by imaging findings. Further evaluation with thoracoabdominal ultrasound identified an avascular cystic mass in the anterior right mediastinum, consistent with a lymphangioma (Figure 5).

As the neonate developed cholestasis secondary to total parenteral nutrition, ursodeoxycholic acid was started at 40 mg twice daily on day 49. The infant was discharged on day 50 and continued follow-up with pediatric surgery and neonatology. At four months of age, surgical correction of the cleft lip was performed. At three years of age, the child demonstrated normal psychomotor development.

## Discussion

Congenital chylothorax, although rare, is the most common cause of congenital pleural effusion in the neonatal period [13]. Its clinical course is heterogeneous. Spontaneous resolution occurs in approximately 22% of cases and is associated with nearly 100% survival [14]. However, when complicated by hydrops fetalis, the condition is associated with high morbidity and mortality, with the latter varying between 22% and 53% [15]. Poor prognostic factors include the presence of congenital malformations, bilateral pleural effusion, hydrops, polyhydramnios, progressive fluid accumulation with mediastinal shift, and early gestational age at diagnosis, in particular, before 30 weeks of gestation [2,16].

Prenatal interventions have been associated with improved outcomes in preterm infants diagnosed with congenital chylothorax, including higher Apgar scores, fewer days of mechanical ventilation, and reduced complication rates in neonates with hydrops fetalis [17,18]. There is currently no consensus regarding the optimal therapeutic approach for these patients, as no randomized controlled trials have been conducted to date, largely due to the rarity and heterogeneity of the condition. Conversely, gestational age at diagnosis, severity, progression of the pleural effusion, and the presence of secondary fetal complications, such as hydrops fetalis, pulmonary hypoplasia, or mediastinal shift, are key determinants in selecting the most appropriate management strategy [8].

Conservative fetal management with close surveillance through serial ultrasound examinations is generally recommended for small to moderate unilateral effusions without signs of hydrops, as spontaneous resolution may occur [19]. In these cases, the prognosis is excellent, with reported survival rates ranging from 73% to 100% [1]. In contrast, cases involving large or progressively worsening pleural effusions or the presence of hydrops fetalis often warrant prenatal intervention. Available options include therapeutic thoracocentesis (single or repeated procedures), PAS, and intrauterine pleurodesis using OK-432 [20-23].

Thoracocentesis is frequently employed as the initial approach due to its combined diagnostic and therapeutic utility; however, its efficacy as a single procedure is limited. In many cases, repeated thoracocentesis or PAS, establishing a continuous drainage pathway between the pleural space and the amniotic cavity, may become necessary for sustained decompression [20,22].

Despite its potential benefits, PAS is associated with a range of complications. Fetal demise following shunt placement has been reported in up to 11% of cases [22]. Catheter migration is frequent, with rates varying between 20% and 65%, and shunt obstruction is also a common concern. Approximately 10-20% of fetal shunts require replacement due to malfunction or displacement [24]. Additionally, PPROM and preterm delivery remain frequent adverse outcomes associated with this intervention [1,2,22,25].

Management decisions must carefully balance the risks and benefits of intervention against the natural course of the effusion. In hydropic fetuses and cases of severe pleural effusion, evidence from larger case series supports the use of invasive treatment, with PAS demonstrating greater efficacy compared to repeated thoracocentesis. In contrast, for non-hydropic fetuses, the benefits of shunting remain less clear [20].

Rustico et al. [1] proposed a management algorithm for primary fetal pleural effusion. In cases diagnosed after 34 weeks’ gestation, immediate thoracocentesis followed by delivery is recommended. Conversely, for mild-to-moderate unilateral effusions without hydrops diagnosed before 34 weeks, weekly ultrasound surveillance is suggested to determine if spontaneous resolution occurs. If the effusion progresses or if hydrops develops, repeated thoracocentesis or PAS should be considered.

Perinatal asphyxia and respiratory insufficiency are major consequences of congenital chylothorax at birth, particularly in cases associated with massive pleural effusion and/or hydrops fetalis [26]. In utero thoracocentesis performed immediately prior to delivery may facilitate adequate lung expansion and reduce the risk of cardiopulmonary compromise following the cessation of umbilical circulation [1,13,26,27]. After birth, neonatal intensive care involves supportive measures, including respiratory and cardiovascular stabilization, pleural drainage, nutritional support, and analgesia [27].

## Conclusions

Our case highlights the critical importance of individualized decision-making within a multidisciplinary fetal medicine center in the management of fetal pleural effusion. This setting enables timely and tailored interventions, such as urgent antepartum thoracocentesis, which have been associated with improved neonatal outcomes, including higher Apgar scores, decreased need for ventilatory support, and reduced postnatal complications. In the present case, perinatal thoracocentesis played a pivotal role in optimizing perinatal outcomes, culminating in the delivery of a live neonate with minimal respiratory morbidity.
